# Bridging the Gap: Challenges and Strategies for the Implementation of Artificial Intelligence-based Clinical Decision Support Systems in Clinical Practice

**DOI:** 10.1055/s-0044-1800729

**Published:** 2025-04-08

**Authors:** Niels Peek, Daniel Capurro, Vlada Rozova, Sabine N van der Veer

**Affiliations:** 1 The Healthcare Improvement Studies Institute (THIS Institute), Department of Public Health and Primary Care, University of Cambridge. Cambridge, UK; 2 Centre for the Digital Transformation of Health, University of Melbourne & The Royal Melbourne Hospital. Melbourne, Australia; 3 Centre for the Digital Transformation of Health, University of Melbourne. Melbourne, Australia; 4 Centre for Health Informatics, Division of Informatics, Imaging and Data Science, Faculty of Biology, Medicine and Health, University of Manchester. Manchester, UK

**Keywords:** Artificial Intelligence, Clinical Decision Support Systems, Implementation Science, Review

## Abstract

**Objectives** : Despite the surge in development of artificial intelligence (AI) algorithms to support clinical decision-making, few of these algorithms are used in practice. We reviewed recent literature on clinical deployment of AI-based clinical decision support systems (AI-CDSS), and assessed the maturity of AI-CDSS implementation research. We also aimed to compare and contrast implementation of rule-based CDSS with implementation of AI-CDSS, and to give recommendations for future research in this area.

**Methods** : We searched PubMed and Scopus for publications in 2022 and 2023 that focused on AI and/or CDSS, health care, and implementation research, and extracted: clinical setting; clinical task; translational research phase; study design; participants; implementation theory, model or framework used; and key findings.

**Results** : We selected and described a total of 31 recent papers addressing implementation of AI-CDSS in clinical practice, categorised into four groups: (i) Implementation theories, frameworks, and models (4 papers); (ii) Stakeholder perspectives (22 papers); (iii) Implementation feasibility (three papers); and (iv) Technical infrastructure (2 papers). Stakeholders saw potential benefits of AI-CDSS, but emphasized the need for a strong evidence base and indicated that systems should fit into clinical workflows. There were clear similarities with rule-based CDSS, but also differences with respect to trust and transparency, knowledge, intellectual property, and regulation.

**Conclusions** : The field of AI-CDSS implementation research is still in its infancy. It can be strengthened by grounding studies in established theories, models and frameworks from implementation science, focusing on the perspectives of stakeholder groups other than healthcare professionals, conducting more real-world implementation feasibility studies, and through development of reusable technical infrastructure that facilitates rapid deployment of AI-CDSS in clinical practice.

## 1. Introduction

Recent years have witnessed a rapid surge in research dedicated to the development of artificial intelligence (AI) algorithms to support clinical decision-making tasks. While many of these algorithms have been crafted with precision and their accuracy validated through rigorous processes, there are concerns that their translation into routine clinical practice remains disproportionately limited [ 1 ]. This gap between algorithm development and clinical deployment not only raises questions about the efficiency of research efforts but also prompts a critical examination of the factors contributing to this translational bottleneck, including the demonstration of clinical effectiveness [ 2 ]. AI algorithms that fail to make their way into clinical practice, despite demonstrating high performance —in terms of accuracy and improving patient outcomes— and validity, can be deemed as research waste. For example, during the COVID-19 pandemic, hundreds of new AI algorithms were developed to support a wide range of aspects of patient care [ 3 ], but only a fraction of these made it into frontline clinical services [ 4 ], mostly operating on a small scale [ 5 ]. The discrepancy between the number of developed and validated algorithms and those integrated into clinical workflows underscores the need to understand the barriers that impede their adoption and explore strategies to enhance their clinical utility.

Translational challenges in healthcare are not unique to AI-based technologies: across areas, there is research continually producing new findings that can contribute to effective and efficient healthcare. However, many of these findings fail to be implemented into routine practice and policy [ 6 ]. And even where new interventions and technologies are deployed in clinical practice, it was preceded by an effortful, unpredictable and typically slow process [ 7 ]. Implementation science is the field that investigates this process, as well as methods to promote the systematic uptake of research findings and other evidence-based practices into routine practice, and, ultimately, to improve the quality and effectiveness of health services [ 8 ].

A common way to implement AI algorithms into practice is by integrating them into clinical decision support systems (CDSS). We will use “AI-CDSS” to refer to CDSS whose outputs are produced by a model or algorithm that was automatically or semi-automatically derived from data, using machine learning (ML) [ 9 ]. These systems excel in capturing subtle patterns in real-world healthcare practice. However, they can also replicate systemic errors and biases, and their decision-making processes are often perceived as “black boxes”, lacking transparency and interpretability [ 10 ]. In contrast, rule-based CDSS are systems that produce outputs by relying on rules and logic provided by human experts [ 11 ]. Historically, most CDSS have been rule-based. The rules that are embedded in these systems are typically based on explicit knowledge and guidelines, making the system interpretable and transparent. However, rule-based systems may struggle to capture the experience-based, tacit knowledge that is often essential to accomplish complex clinical tasks. The choice between rule-based CDSS and AI-CDSS often depends on the specific clinical context, available data, expertise, and the need for transparency in decision-making.

Many rule-based CDSS have been deployed in clinical practice, and the process of implementing these systems has been well studied. For instance, Miller et al. [ 12 ] conducted a review of qualitative studies published between 2000 and 2013 that investigated the experiences of healthcare professionals with rule-based CDSS. They found that clinician-patient-system integration, system usability, algorithmic refinement, system maturity, and patient safety were critical themes. Their findings highlighted the necessity of understanding the intricate interaction dynamics between human decision-makers and CDSS. Liberati et al. [ 13 ] investigated barriers and facilitators to the uptake of rule-based CDSS across diverse health professionals in hospitals at different stages of CDSS adoption. Their results underscored the dynamic nature of barriers and facilitators, and identified factors such as clinicians' attitudes toward scientific evidence, the quality of interdisciplinary relationships, and organisational transparency and accountability as critical elements influencing the readiness of hospitals to adopt CDSS. Meunier et al. [ 14 ] reviewed 48 studies focusing on the use of CDSS by primary care providers. They found that increased workload is the greatest barrier to using CDSS in clinical practice, in addition to further human, organizational, and technological factors that may negatively affect the adoption of CDSS.

While it is plausible that some of the lessons learned about rule-based CDSS implementation will transfer to AI-CDSS, it is equally likely that there will be differences. The current narrative review therefore aimed to: (i) provide a narrative review of recent literature on implementation of CDSS in clinical practice, focusing specifically on AI-CDSS, i.e. systems that are based on machine learning; (ii) assess the maturity of AI-CDSS implementation research; (iii) compare and contrast what is known about implementation of rule-based CDSS with what is known about the implementation of AI-CDSS; and (iv) to give recommendations for future research on implementation of AI-CDSS.

## 2. Methods

Building on the same search syntax used by Hogg et al. in their recent review of theories, models and frameworks used in healthcare AI implementation research [ 15 ], we searched Medline (via PubMed) and Scopus for publications since October 2022 that focused on AI and/or CDSS, healthcare, and implementation research. We also conducted a forward (i.e., ‘cited by’) and ‘similar article’ search in PubMed for a set of recent, seminal papers [ 15 16 17 18 19 20 21 ], and manually searched included papers in recent systematic reviews of the literature on this topic [ 14 , 15 , 20 21 22 23 24 25 26 27 28 ]. We aspired to give a comprehensive, but not exhaustive, picture of the recent literature on implementation of AI-CDSS. We only included original research papers and systematic or scoping literature reviews, while excluding study protocols, editorials, commentaries and narrative reviews. We excluded studies that evaluated CDSS usability, because usability evaluation is typically conducted prior to system implementation. We also excluded research that exclusively focused on: AI development but without describing an implementation effort; autonomous AI (i.e., not involving human decision makers); and on assessing the impact of AI-CDSS on health outcomes.

**Figure 1.**
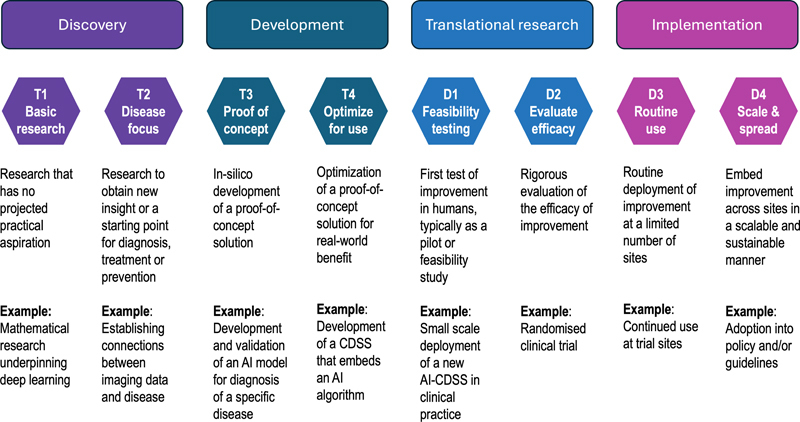
The steps from translatable to translational research as defined by Gannon [ 30 ], with examples of AI-CDSS research.

From included papers, we extracted the clinical setting in which the AI-CDSS was deployed (or considered for deployment), the clinical task which it intended to support, the translational research phase as reported in the paper, the study design, the type and number of study participants, and the implementation theory, model or framework that was used to guide study methods and/or interpret the findings. To describe translational research phases, we will use Gannon's [ 30 ] conceptual model for translatable and translational research ( Figure 1 ), which ranges from basic research that has no projected practical (clinical practice or commercial) aspiration to large scale, sustainable deployment of healthcare improvements. We analysed extracted data thematically and provided a theme-level synthesis of findings across studies if the number and type of studies within a theme allowed this.

## 3. Results

**Figure 2.**
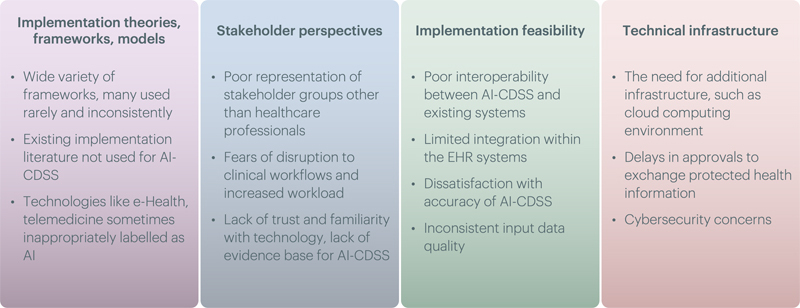
Overview and summary of the four groups of papers emerging from the survey.

We included 31 recent papers addressing implementation of AI-CDSS in clinical practice. We categorised these papers into four groups ( Figure 2 ): (i) Studies presenting new theories, frameworks, and models for AI-CDSS implementation (four papers); (ii) Studies assessing stakeholder perspectives on AI-CDSS implementation (22 papers); (iii) Studies evaluating the implementation feasibility of AI-CDSS (three papers); and (iv) Studies presenting technical infrastructure for implementing AI-CDSS (two papers). Below we discuss each group in more detail.

### 3.1. Implementation Theories, Frameworks, and Models

An important aspect of implementation science is the application of theories, models and frameworks to inform and study implementation processes [ 31 ]. A variety of theories, models and frameworks have been proposed in the literature; one of the more recent, widely used and comprehensive frameworks is the Consolidated Framework for Implementation Research (CFIR) [ 32 ], which aims to predict or explain barriers and facilitators to implementation effectiveness. Implementation theories, models and frameworks offer an efficient way of generalizing findings across diverse healthcare settings, and thus of consolidating the learnings from individual studies. Conversely, they can play a key role in the design of implementation studies, helping to identify potential barriers and facilitators to successful implementation, guide the selection of implementation strategies, and helping to frame study questions, motivate hypotheses, and contextualize results. We discuss three recent papers specifically focusing on the role of theories, models and frameworks in AI-CDSS implementation research.

Hogg et al. [ 15 ] aimed to characterise the application of theories, models and frameworks in clinical CDSS research, specifically focusing on qualitative studies. They found 202 studies published between January 2014 and October 2022. The type of CDSS studied was rule-based in 88 studies (43.6%), based on AI in 98 studies (48.5%), and not specified in 16 studies (7.9%). Seventy studies (34.7%) applied an implementation theory, model or framework. There was an eightfold increase in the number of publications between 2014 and 2022 but no increase in the proportion applying theories, models or frameworks. Of the 50 theories, models, and frameworks applied, 40 (80%) were only applied once. The Technology Acceptance Model (TAM) [ 33 ], published in 1989 and considered outdated since the 2003 publication of its successor (Unified Theory of Acceptance and Use of Technology [UTAUT] [ 34 ]), was applied most frequently (n=9), followed by UTAUT and CFIR, which were each applied seven times.

Also Gama et al. [ 27 ] conducted a review of the literature, specifically aiming to identify implementation frameworks used to understand the application of AI-CDSS in healthcare practice; they identified seven studies. Their literature search revealed that many technologies (for instance, eHealth and telemedicine) are currently inappropriately labelled as AI. This probably reflects the hype surrounding AI and the tendency to adopt fashionable terms to increase attention, readership, and chances of publication. But this type of misuse of AI terminology does create ambiguity and confusion for researchers attempting to synthesize learning in this field. A second key finding was that none of the identified studies referred to the existing implementation literature to inform their data analysis or framework development. This is quite surprising, given the recognition of the challenges of AI-CDSS implementation. Although AI-CDSS are likely to have additional requirements that are not relevant for other interventions, there is a wealth of literature on implementation challenges and facilitators that could inform the AI field and accelerate learning. The authors concluded that our understanding of how to implement AI-CDSS in healthcare practice is still in its early stages of development.

Drawing on practices from applied systems engineering, software engineering, and health care ML software development, Assadi et al. [ 29 ] present a framework for clinical AI model implementation that identifies four phases: i) Inception, ii) Preparation, iii) Development, and iv) Integration. Each phase incorporates considerations from the domains of integration and systems engineering as well as the interaction between them for an integrated “system of systems”, i.e. a system that is composed of other systems and its elements are managerially and/or operationally independent. For each phase, they present specific elements for consideration in three domains of integration: the human, the technical system, and the environment. Although this shows that Assadi's framework goes beyond technical integration, its underlying engineering and software development principles make it particularly relevant for informing the design and development of the technical infrastructures for deploying AI-CDSS in practice (see also ‘Technical infrastructure’ below).

Lastly, Van der Vegt et al. [ 19 ] derived an end-to-end implementation framework for AI-CDSS, called SALIENT, based on the taxonomy of Stead et al. for translating informatics interventions from lab to field [ 35 ], integrated with reporting standards for AI research (TRIPOD [ 36 , 37 ], DECIDE-AI [ 38 ], CONSORT-AI [ 39 ]), and further refined after review of 20 published clinical AI implementation frameworks. SALIENT aims to comprehensively address the what (components), when (stages), and how (tasks) of AI implementation, as well as the who (organization) and why (policy domains); it currently still requires validation to ensure its applicability to real-world studies of deployed AI-CDSS.

### 3.2. Stakeholder perspectives

A large number of studies assessed factors that may positively or negatively affect the implementation and adoption of AI-CDSS in healthcare settings. These factors are typically identified by interviewing and surveying relevant stakeholders, such as physicians, nurses and other healthcare professionals, healthcare information technology (HIT) specialists, AI and CDSS researchers, and others. Studies can be conducted prior to implementing a system (to inform design and prepare for its deployment), after implementing a system, or independently of a specific system (e.g., to explore stakeholders' perceived implementation barriers for AI-CDSS in general). Ideally, such studies use implementation theories, models and frameworks to guide study design, data collection and analysis, and interpretation of findings.

Table 1 lists five recently published reviews assessing stakeholder perceptions of CDSS [ 14 , 21 , 23 , 25 , 28 ]. From these, two reviews [ 14 , 23 ] included only or mostly rule-based CDSS, two reviews [ 25 , 28 ] exclusively focused on AI-CDSS, and one review [ 21 ] included both rule-based and AI-CDSS implementation studies. The two AI-CDSS reviews focused on specialist settings (radiology and pathology) while the other three had a broader perspective. Two reviews [ 14 , 25 ] were limited to studies involving healthcare professionals (HCPs) and medical students. While the other reviews included any stakeholder group, most eligible studies focused on HCPs. This point was specifically emphasized by Hogg et al. [ 21 ], who found that 70% of the findings across eligible studies came from HCPs, while other stakeholder groups made much smaller contributions (patients, carers and other members of the public, 11.4%; developers, 7.7%; health care managers and leaders, 7.5%; regulators and policy makers, 3.4%). Across the five reviews, disruption to clinical workflows and increased workload were often identified as key barriers to CDSS implementation, while perceived usefulness of CDSS outputs was often identified as a key facilitator. Further factors mentioned were lack of awareness, knowledge, trust, and familiarity with the technology; technical dependencies and design; quality of input data; evidence base for AI-CDSS; contextual fit of CDSS with users' roles/clinical setting; and perceived threat to professional autonomy. The review by Hogg et al. [ 21 ] specifically compared rule-based CDSS with AI-CDSS and found that most influencing factors for implementation of rule-based CDSS also applied to AI-CDSS, except for intellectual property, regulation, and sociocultural attitudes.

**Table 1.** Overview of reviews published in 2023 assessing stakeholder perceptions of rule-based and/or AI-CDSS.

First author / reference	Primary review aim	Review type	Setting	Stakeholder groups of interest	Theory, model or framework	Included studies (AI-CDSS/rule-based)	Key findings
Meunier [ 14 ]	Identify and quantify barriers and facilitators to CDSS	Mixed methods systematic review	Primary care	Primary care professionals (at least 50% of study sample)	HOT-fit	48 (0/48)	Qualitatively, barriers and facilitators were classified as human (e.g., perceived usefulness), organizational (e.g., disruption of usual workflow), and technological (e.g., CDSS usability). Increased workload was the greatest barrier to using CDSS in clinical practice. Quantitatively, human and organizational factors had negative impacts on CDSS use, whereas technological factors had no impact.
Abell [ 23 ]	Identify, categorize, and describe barriers and facilitators to CDSS implementation	Mixed methods scoping review	Hospitals (inpatient and outpatient)	Any	NASSS	44 (2/42)	Participants in most studies (n=40) had clinical or patient-facing roles. The most common influencing factors included: fit of CDSS with workflows (n=19]; usefulness of CDSS output in practice (n=17]; CDSS technical dependencies and design (n=16); users' trust in CDSS input data and evidence base (n=15); and contextual fit of CDSS with users' roles/clinical setting (n=14).
Hogg [ 21 ]	Identify key stakeholders, consolidate their perspectives on clinical AI implementation	Qualitative evidence synthesis	Any	Any	NASSS	111 (41/66) *	Five distinct stakeholder groups: HCPs; patients, carers, and other members of the public; AI-CDSS developers; health care managers and leaders; and regulators and policy makers. HCPs contributed 70%, whereas only 3% came from regulators and policy makers. Most influencing factors for implementation of rule-based CDSS also applied to AI-CDSS, except for intellectual property, regulation, and sociocultural attitudes.
Eltawil [ 25 ]	Determine barriers and enablers for acceptance of AI-CDSS	Mixed methods scoping review	Radiology	Physicians and medical students	None reported	12 (12/0)	Barriers were: lack of awareness, knowledge, trust, and familiarity with the technology; unstructured implementation processes; no confidence benefits of AI-CDSS would translate into improved patient outcomes; and perceived threat to professional autonomy. Where HCPs expected AI-CDSS to have high clinical potential, this acted as an enabler. Areas of expected clinical potential included: fewer diagnostic errors; increased diagnostic efficiency; and improved quality of care.
King [ 28 ]	Determine contextual factors that may support or constrain the uptake of AI-CDSS	Realist review	Pathology	Any	NPT; IPOE model **	101 (101/0)	Uptake of AI-CDSS in pathology requires measures that either increase confidence in the system or provide users with an understanding of the system's performance. For specialist centres, efforts should focus on reducing workload rather than increasing accuracy. Designers also need to give careful thought to usability and how AI-CDSS is integrated into workflows.

**Abbreviations:** AI, artificial intelligence; AI-CDSS, Artificial Intelligence-based clinical decision support system; CDSS, clinical decision support system; HCPs, Health care professionals; HOT-fit, Human, Organizational, Technology, Net Benefits framework [ 62 ]; IPOE model, input-process-output-engage model [ 63 ]; NASSS, Nonadoption, Abandonment, Scale-up, Spread and Sustainability (NASSS) framework [ 61 ]; NPT, Normalisation Process Theory [ 64 ].

* For four studies, the type of CDSS was not specified

** The review considered a range of theories and frameworks but mentioned Normalisation Process Theory (NPT) and the input-process-output-engage (IPOE) model as particularly useful

We have also summarized 17 recent studies [ 16 , 17 , 40 41 42 43 44 45 46 47 48 49 50 51 52 , 54 , 65 ] reporting original research assessing stakeholder perspectives of AI-CDSS in Table 2 . These studies appeared after the five reviews listed in Table 1 were completed. From these 17 studies, most focused on screening, diagnosis, or prediction tasks in hospital settings. Only seven studies (41%) used an implementation theory, model or framework, with three of those studies using CFIR (one study used UTAUT; no study used TAM). The potential added value of AI-CDSS, with opportunities for quality improvement and time saving, was repeatedly mentioned as a facilitating factor for the adoption of AI-CDSS in clinical settings. Potential barriers that emerged across studies were (lack of) trust in, and transparency of, AI systems; pressures on the time of clinical staff and the increased workload associated with a new system; the limited knowledge of AI among HCPs and hence the need for education and training; the need for a strong evidence base for AI-CDSS; poor system interoperability; risk of errors; and poor usability and workflow integration.

**Table 2.** Overview of recent studies assessing stakeholder perceptions of AI-CDSS.

Article	Country	Setting	Clinical task	Translational research phase *	Study design	Participants (type and number)	Theory, model or framework	Key findings
Bergquist et al. [ 45 ]	Sweden	Clinical radiology	No specific clinical task	Not linked to a specific system	Interviews	Radiologists; managers; other medical professionals; engineers (n=25)	None reported	Key requirements for the adoption of AI-CDSS in clinical radiology: trust in relation to reliability, transparency, quality verification, and inter-organizational compatibility.
Fazakarley et al. [ 46 ]	UK	Hospital	Diagnosis of CAD based on stress echo-cardiography	T2	Interviews	Doctors; nurses; HIT experts; researchers (n=13)	None reported	Participants were generally open to and optimistic about the use of AI but concerned about the security of patient data, the potential for misdiagnosis, and increased workload.
Fujimori et al. [ 16 ]	Japan	Emergency department	Predicting the risk of aortic dissection	D4	Mixed-methods lab study with clinical vignettes	Resident physicians; emergency physicians (n=14)	UTAUT; CFIR	Key facilitators were evidence strength (size of the dataset used to train the AI model) and system design quality. Main barrier was lack of relative advantage for typical cases coupled with the potential ability to bias physicians' decision making.
Hesso et al. [ 47 ]	UK	Hospital	Cancer diagnosis based on medical imaging	Not linked to a specific system	Survey, followed by interviews	HCPs involved in lung, breast, colorectal or prostate cancer care (n=95)	None reported	Participants agreed that the use of AI would enhance the care pathway for cancer patients. The majority (73%) of respondents had never utilised AI. It was felt that there is need for education and training of HCPs in AI.
Ho et al. [ 48 ]	US	Primary care	Screening for peripheral arterial disease	D4	Interviews	Primary care physicians; cardiovascular specialists; patients (n=26)	CFIR	Physicians felt that a diagnostic AI-CDSS would improve patient care but cited limited time and authority in asking patients to undergo additional screening procedures. Patients were interested in having their physicians use the tool but raised concerns about AI replacing human decision-making.
Manetti et al. [ 49 ]	Italy	Hospital in-patient care	Early detection of sepsis	D4	Interviews	Nurses; non-nursing professionals (n=25)	None reported	Organizational redesign was identified as the primary adoption driver. Even though nurses perceived workload increase related to the AI-CDSS, technology acceptability was relatively high, as the standardization of tasks was perceived to be crucial for improving professional satisfaction.
Neher et al. [ 17 ]	Sweden	No specific setting	No specific clinical task	Not linked to a specific system	Interviews	Healthcare leaders (n=26)	CFIR	Participants saw clear potential benefits of AI-CDSS and believed it to be more effective and precise in certain cases. They questioned the evidence base behind AI-CDSS technology, its transparency, potential quality improvement, and safety risks and expressed uncertainty about the adaptability and trialability of AI. Complexities such as the characteristics of the technology, the lack of conceptual consensus about AI, and the need for a variety of implementation strategies to accomplish transformative change in practice were identified, as were uncertainties about the costs involved in AI implementation.
Petersson et al. [ 50 ]	Sweden	No specific setting	No specific clinical task	Not linked to a specific system	Interviews	Healthcare leaders (n=26)	None reported	Three types of challenge were perceived to be linked with the implementation of AI-CDSS in healthcare: 1) Conditions external to the healthcare system; 2) Capacity for strategic change management; 3) Transformation of healthcare professions and healthcare practice.
Pumplun et al. [ 51 ]	Germany	No specific setting	Diagnosis	Not linked to a specific system	Interviews	Physicians; HIT supplier staff (n=22)	NASSS	The authors established an integrated overview of factors specific to AI-CDSS adoption in clinical practice and create an operationalised maturity model that healthcare provider organisations can apply to assess their current state of adoption progress to decide on further actions and prioritise investments.
Redrup Hill et al. [ 52 ]	UK	No specific setting (but using pathology as exemplar context)	Diagnosis	Not linked to a specific system	Online workshops	Software developers; patients; healthcare professionals; regulators (n=31)	Beauchamp and Childress's Four Principles [ 53 ]	The authors identified ethical and legal factors relevant for considering the type and level of human involvement when implementing AI tools to support diagnosis. They grouped these into six themes: risk and potential harms; impacts on human experts; equity and bias; transparency and oversight; patient information and choice; accountability, moral responsibility and liability for error. They concluded that, although these factors will be relevant across areas of healthcare, the implications of these factors will be highly context-specific.
Schepart et al. [ 54 ]	US	Cardio-vascular medicine	No specific clinical task	Not linked to a specific system	Interviews, followed by survey	Cardiologists; HIT administrators (interviews n=20; survey n=120)	None reported	The authors identified 5 major challenges: (1) limited knowledge, (2) insufficient usability, (3) cost constraints, (4) poor EHR interoperability, and (5) lack of trust. A minority of cardiologists were using AI tools; more were prepared to implement AI tools, but their sophistication level varied greatly.
Schwartz et al. [ 65 ]	US	Hospital in-patient care	Predicting in-patient deterioration	T1	Interviews	Nurses; physicians; physician assistants; nurse practitioners (n=17)	Madsen & Gregor's human-computer trust conceptual framework [ 66 ]	Perceived understandability and perceived technical competence (i.e. CDSS accuracy) influence clinicians' trust in predictive CDSS. Additional relevant factors are evidence, perceived actionability, and equitability. There were profession-specific factors characterising the relationship between understandability and trust. Perceptions of trust were largely the same between nurses and prescribing providers.
Strohm et al. [ 40 ]	The Netherlands	Radiology	Bone maturity assessments based on X-rays of paediatric patients' hands	T1	Interviews	Radiologists; innovation managers; data scientists; junior physicians (n=24)	NASSS	Facilitating factors were: pressures for healthcare cost containment; high expectations of AI's added value; presence of hospital-wide innovation strategies; and presence of a “local champion”. Hindering factors were: inconsistent technical performance of AI applications; unstructured implementation processes; uncertain added value for clinical practice of AI applications; and large variance in acceptance and trust among direct (radiologists) and indirect (referring clinicians) users.
Terry et al. [ 41 ]	Canada	Primary care	No specific clinical task	Not linked to a specific system	Interviews	Primary healthcare practitioners; decision makers; researchers (n=14)	None reported	Participants viewed AI with a guarded but hopeful stance. They emphasized that AI tools relevant to the needs of practitioners need to be developed. Main concerns were impact on clinical skills, introduction of errors, and loss of control in decision-making. Ethical, legal, and social considerations included: medical-legal issues, potential biases, equity, lack of transparency, loss of control over data, and privacy and security of data. Necessary foundational elements to support the uptake of AI tools included: co-creation, high-quality training data, and rigorous evaluation.
Van der Meijden et al. [ 42 ]	The Netherlands	Intensive care	Prediction of readmission and mortality risk after ICU discharge	D4	Survey	Physicians (n=64)	None reported	Most participants were familiar with AI and had positive expectations about it. Not all physicians found the decision to discharge a patient complex, yet nearly all agreed that a discharge CDSS could be of value. Physicians at the site where the AI-CDSS tool was developed showed greater familiarity with AI and had a stronger belief in the supportive role of AI in general and in the target CDSS. Other physicians attached more importance to understanding which factors contributed to the predictions.
Wang et al. [ 43 ]	US	Hospital	Detection of peripheral arterial disease	T1	Interviews	Technical, administrative, and clinical staff interacting with the AI-CDSS (n=12)	None reported	Positive translational factors included strong clinical leadership, trustworthy workflows, early consideration of end-user needs, and ensuring that the CDSS addressed an actionable problem. Negative factors included failure to incorporate the on-the-ground context, the lack of feedback loops, and data silos limiting the AI-CDSS.
Weinert et al. [ 44 ]	Germany	Hospital	No specific clinical task	Not linked to a specific system	Survey	Chief information officers; HIT professionals; data scientists (n=40)	None reported	Most participants recognised the implementation of AI as a relevant, forthcoming part of their IT strategy. Time-saving effects, competitive advantage, and increase in care quality were seen as key opportunities associated with AI. Lack of resources, poor interoperability with the existing IT infrastructure, staffing resources, time, knowledge, financial resources, and technical resources were viewed as potential barriers.

**Abbreviations:** AI, artificial intelligence; CAD, coronary artery disease; CDSS, computerised decision support system; CFIR, Consolidated Framework for Implementation Research; EHR, electronic health record; HCP, healthcare professional; HIT, healthcare information technology; ICU, intensive care unit; IT, information technology; NASSS, Non-adoption, Abandonment, Scale-up, Spread, and Sustainability; UTAUT, Unified Theory of Acceptance and Use of Technology.

* See Figure 1 for translational research phases as defined by Gannon [ 30 ].

### 3.3. Implementation feasibility

The third group that we identified consisted of papers reporting real-world assessment of AI-CDSS feasibility, typically based on a pilot implementation in clinical practice.

Petitgand et al. [ 55 ] reported on the pilot implementation of an AI-CDSS in the emergency department of a large academic health centre in Canada. Presenting patients were asked to complete a questionnaire on a mobile tablet, from which the CDSS extracted their chief complaint, medical history, and identified red flags and signs of serious conditions. Due to poor interoperability between the AI-CDSS and other clinical information systems, CDSS outputs had to be printed and handed to physicians in paper form, which did not always happen. Physicians reported that the system was good at reporting simple complaints (a localized pain, a broken leg, etc.) but poor at making sense of multi-complaint conditions, which applies to most patients presenting at emergency departments. Also, some physicians reported having discovered errors in the medical histories, which then led them down the wrong diagnostic path. As a result of these issues, physician adoption rates were only around 30%. When adjustments were made to the system in response to the issues raised, adoption rates did not increase.

Romero-Brufau et al. [ 18 ] conducted a survey among physicians, nurses and social workers in three primary care outpatient clinics, before and after implementation of a commercial AI-CDSS aiming to improve glycemic control in diabetes patients. The CDSS identified patients at risk for poor glycemic control and generated intervention recommendations to reduce that risk. The system used a combination of data from the electronic health records (EHR) and further social determinants of health. Although the data was imported directly from the EHR, the risk calculator and recommendation delivery were stand-alone tools that were not integrated within the EHR due to design and interoperability limitations. Staff completed 45 surveys before the implementation and 38 after the implementation. Following implementation, staff felt that care was better coordinated but only 14% of users would recommend the AI-CDSS, with recommended interventions often being considered inadequate. A favourable aspect of the CDSS was that it promoted team dialog about patient needs.

Smak Gregoor et al. [ 56 ] conducted a mixed-methods pilot feasibility study with a commercial mHealth app for skin lesion assessment, implemented in primary care. Patients who contacted their general practitioner (GP) because of a suspicious skin lesion were asked to use the app to classify the lesion as high or low risk for skin cancer based on smartphone pictures. Fifty patients were recruited, of whom 42 (84%) completed the skin lesion assessment via the app. Although GPs never changed their working diagnosis, they sometimes did change their treatment plan based on the app's assessments. Notably, 54% of patients with a benign skin lesion and low risk rating indicated that they would be reassured and cancel their GP visit considering these results. The authors concluded that implementation of an AI-based mHealth app for detection of skin cancer in primary care appeared feasible.

### 3.4. Technical infrastructure

The final theme consisted of papers presenting new technical infrastructure to facilitate the deployment of AI-CDSS in clinical practice. Tseng et al. [ 57 ] developed a new process that standardizes health care information for assessing inpatient deterioration detection based on vital signs. They present a technical implementation guide that includes Health Level 7 Fast Healthcare Interoperability Resources (HL7 FHIR) data mapping, a system architecture, a workflow, and FHIR applications. Afshar et al. [ 58 ] developed a cloud service designed to ingest, process, and store clinical notes as HL7 messages from the Epic EHR using natural language processing in an elastic cloud computing environment. The service was used to implement a deep learning algorithm for screening for opioid misuse at the University of Wisconsin Hospital across the surgical and medical hospital inpatient wards. The longest delay in pipeline development was because of cybersecurity approvals, especially because of the exchange of protected health information between the Microsoft and Epic cloud vendors. In silent testing, the resultant pipeline provided a computerised alert to the bedside within minutes of a provider entering a note in the EHR.

## 4. Discussion

### 4.1. Summary of findings

A well-known implementation gap, coined as the “AI chasm” [ 59 ] or “last mile problem” [ 60 ], is preventing AI from realising its potential benefits in real-world clinical practice. We reviewed recent literature on implementation of CDSS, focusing specifically on AI-CDSS, i.e. systems that are based on machine learning. Many of the 31 selected papers focused on assessing stakeholder perspectives on factors influencing implementation and adoption of AI and AI-CDSS. Stakeholders clearly saw potential benefits of AI-CDSS, but emphasized the need for a strong evidence base for AI-CDSS and indicated that systems should seamlessly fit into clinical workflows. There were concerns around trust and transparency; limited knowledge of AI among HCPs; poor system interoperability; and risk of errors. Many factors that stakeholders perceived as influential for the implementation of rule-based CDSS also applied to AI-CDSS, but there were also differences regarding trust and transparency, knowledge, intellectual property, and regulation. We found that existing theories, models and frameworks from the field of implementation science were rarely and inconsistently used in AI-CDSS implementation research. When they were, there appeared to be a trend towards using UTAUT, CFIR, and the Nonadoption, Abandonment, Scale-up, Spread and Sustainability (NASSS) framework [ 61 ], but there was limited description of selection rationale and a lack of clarity on how the framework informed the research. In feasibility studies clinician adoption rates were sometimes poor, due to poor system integration and dissatisfaction with accuracy and usefulness of CDSS outputs. Favourable results were obtained in a pilot study with a skin lesion assessment app in primary care [ 56 ], perhaps because it reduced the number of consultations needed for low risk lesions.

### 4.2. Interpretation

The field of AI-CDSS implementation research is still in its earliest stages of development. Stakeholder perspectives have been reasonably well investigated, but there has been a much stronger emphasis on gathering the perspectives of HCPs than other stakeholder groups. Regulators and policy makers were particularly poorly represented. This underrepresentation of perspectives from stakeholders other than HCPs may limit the anticipation and management of the factors that influence successful implementation. Also, relatively few studies have obtained stakeholder perspectives following the implementation of AI-CDSS. When they did, sometimes serious issues with respect to accuracy and usefulness of CDSS outputs emerged and clinician adoption rates were poor – essentially, the implementation had failed.

A second symptom of relative immaturity of the field is the poor use of the existing theories, models, and framework. There exists a rich literature on implementation theories, models, and frameworks from which AI-CDSS implementation research can benefit. But these are rarely and inconsistently used in AI-CDSS implementation research, and sometimes poorly chosen. At the positive side, there seems to be a trend towards using two well established and frequently used frameworks, CFIR [ 32 ] and NASSS [ 61 ]. If the trend persists, this will increasingly help to consolidate and generalise learning from individual studies and solidify the evidence base for AI-CDSS implementation.

Rule-based CDSS have found their way to clinical practice in the last 25 years, which has provided a rich literature on implementation efforts with these systems. There are clearly lessons for AI-CDSS to be learned from that literature. Most of the human and contextual factors relevant to rule-based CDSS (system usability; clinical workflow integration; time pressures on clinical staff; and so on) seem equally relevant to AI-CDSS. But there are also differences. For instance, the concept of trust is intimately related to AI. Rule-based CDSS are typically based on well-established guidelines or quality standards, and therefore there is no reason, from a clinical perspective, not to trust the system outputs. Historically, trust has therefore not been a prevalent theme in the CDSS literature. This has changed in recent years with the advent of AI-CDSS.

In comparison to the efforts to develop AI models underlying AI-CDSS, the technical infrastructure required to deploy AI-CDSS has been minimally explored. This presents significant challenges for hospitals and health services considering adopting them. It is easy to see that the implementation of AI-CDSS faces a “precedence paradox”: there is a need to generate evidence about their clinical impact, but this requires existing infrastructure and implementation pathways in those clinical settings. However, these are frequently not available and health organisations might not want to invest due to the lack of clinical evidence of their efficacy.

### 4.3. Recommendations

There is a clear opportunity to strengthen AI-CDSS implementation research by firmly grounding AI-CDSS implementation studies in established theories, models and frameworks from the field of implementation science. This will not only facilitate the interpretation of findings in individual studies but also help to consolidate and generalize learnings across studies. There may be merit in developing and utilizing theories, models or frameworks specific to AI-CDSS implementation (such as the SALIENT framework [ 19 ]).

We believe that there is no need to further investigate the perspective of HCPs on AI-CDSS, especially not in pre-implementation phases – this perspective has been studied extensively already. There is still a need to better understand the perspectives of other stakeholder groups, such as patients, carers, and other members of the public; AI-CDSS developers; health care managers and leaders; and especially regulators and policy makers. It will also be useful to capture the perspectives of HCPs subsequent to the implementation of AI-CDSS.

More studies are needed that assess feasibility of AI-CDSS in clinical practice and capture the human, technical, and organisational challenges that emerge in such real-world deployments. It is particularly important that the findings from such studies inform future development of AI-CDSS, their deployment in clinical practice, and implementation studies.

Finally, to break the deadlock that results from the “precedence paradox”, major efforts should be targeted towards the design, development, and investigation of reusable technical infrastructure that facilitates rapid deployment and validation of AI-CDSS in clinical practice – consisting, for instance, of HL7 FHIR interfaces to feed EHR data into AI models and user interface templates for CDSS outputs, underpinned by the required information governance approvals and cybersecurity checks. This will help healthcare organisations to assess feasibility and generate evidence of impact more easily, without having to make major infrastructural investments for each pilot. It should be a major area of investment in the near future, ideally with involvement from EHR vendors (for example, see [ 67 ]).

### 4.4. Limitations

Our review has a number of limitations. We did not perform an exhaustive literature search, and may therefore have missed some relevant papers. However, due to the extensive forward and backward searching from recent seminal papers, including several reviews, we believe that we have included the majority of relevant publications. Many authors were vague about their definitions of AI and CDSS, and therefore it was not always clear whether their work was relevant for our review. Similarly, the distinction between rule-based and AI-CDSS was not always made. For these reasons, we may have included or excluded papers inappropriately, or incorrectly labelled systems as rule-based or AI-CDSS. We only actively searched for recently published studies and reviews (published since October 2022), making it hard to assess trends over time. Yet we did include some older papers (from 2019-2022) that were identified via backward searching, and all included reviews covered significantly longer time periods – often going back at least a decade. Most of the AI-CDSS that we reviewed were supporting diagnosis or prediction tasks, while few addressed other clinical decision-making tasks such as prescribing or test ordering. This may have been a side effect of our search strategy. Finally, for pragmatic reasons we excluded studies that only focused on evaluating the impact of AI-CDSS on clinician behaviour and/or patient outcomes. It is conceivable that some of these studies would shed light on implementation of AI-CDSS as well.

## 5. Conclusion

Despite the high expectations surrounding AI-CDSS in healthcare, research has predominantly been technology-centric rather than focused on the changes required for successful deployment of this technology in clinical practice. To date, most AI-CDSS implementation studies have focused on gathering perspectives of HCPs prior to actual deployment in clinical practice. HCPs saw potential benefits of AI-CDSS, but emphasized the need for a strong evidence base and indicated that systems should seamlessly fit into clinical workflows. There were many similarities with rule-based CDSS, but also differences with respect to trust and transparency, knowledge, intellectual property, and regulation. The field can be strengthened by grounding AI-CDSS implementation studies in established theories, models and frameworks from implementation science, focusing on the perspectives of other stakeholder groups than HCPs, conducting more real-world implementation feasibility studies, and through development of reusable technical infrastructure that facilitates rapid deployment of AI-CDSS in clinical practice.
